# Sending or Not-Sending Twin-Field Quantum Key Distribution with Flawed and Leaky Sources

**DOI:** 10.3390/e23091103

**Published:** 2021-08-25

**Authors:** Yi-Fei Lu, Yang Wang, Mu-Sheng Jiang, Xiao-Xu Zhang, Fan Liu, Hong-Wei Li, Chun Zhou, Shi-Biao Tang, Jia-Yong Wang, Wan-Su Bao

**Affiliations:** 1 Henan Key Laboratory of Quantum Information and Cryptography, SSF IEU, Zhengzhou 450001, China; lyf@qiclab.cn (Y.-F.L.); jms@qiclab.cn (M.-S.J.); zxx@qiclab.cn (X.-X.Z.); lf@qiclab.cn (F.L.); lhw@qiclab.cn (H.-W.L.); zc@qiclab.cn (C.Z.); 2 Synergetic Innovation Center of Quantum Information and Quantum Physics, University of Science and Technology of China, Hefei 230026, China; 3 National Laboratory of Solid State Microstructures, School of Physics and Collaborative Innovation Center of Advanced Microstructures, Nanjing University, Nanjing 210093, China; 4 QuantumCTek Co., Ltd., Hefei 230088, China; tangsb@ustc.edu.cn; 5 CAS Quantum Network Co., Ltd., Shanghai 201315, China; wangjiayong@qtict.com

**Keywords:** twin-field, practical security, loss-tolerant

## Abstract

Twin-field quantum key distribution (TF-QKD) has attracted considerable attention and developed rapidly due to its ability to surpass the fundamental rate-distance limit of QKD. However, the device imperfections may compromise its practical implementations. The goal of this paper is to make it robust against the state preparation flaws (SPFs) and side channels at the light source. We adopt the sending or not-sending (SNS) TF-QKD protocol to accommodate the SPFs and multiple optical modes in the emitted states. We analyze that the flaws of the phase modulation can be overcome by regarding the deviation of the phase as phase noise and eliminating it with the post-selection of phase. To overcome the side channels, we extend the generalized loss-tolerant (GLT) method to the four-intensity decoy-state SNS protocol. Remarkably, by decomposing of the two-mode single-photon states, the phase error rate can be estimated with only four parameters. The practical security of the SNS protocol with flawed and leaky source can be guaranteed. Our results might constitute a crucial step towards guaranteeing the practical implementation of the SNS protocol.

## 1. Introduction

Quantum key distribution (QKD) promises to share a common secret key with its security guaranteed by the principles of quantum physics [1]. Notable progress has been made towards QKD over the practical security and performance [2,3]. The decoy-state method [4,5,6] allows the use of the practical sources while maintaining the secret key rate (SKR) at a level comparable to that of the perfect single-photon source. The measurement-device-independent QKD (MDI-QKD) [7] can remove all potential security loopholes in the photon-detection unit.

However, the fundamental limits [8,9] indicate that the SKR scales linearly with the transmittance of channel between Alice and Bob in the absence of the quantum repeater. Fortunately, remarkable progress has been made with the proposal of twin-field QKD (TF-QKD) [10] and its variants [11,12,13,14,15,16]. TF-QKD protocols improve the SKR to the square root of the channel transmittance, which means it can surpass the repeaterless secret key capacities [8,9]. In addition, TF-QKD protocols possess the property of measurement-device-independent and can be applied using the practical light source (e.g., the coherent light source) with the decoy-state method. It is equivalent to prepare the two-mode single-photon states with the coherent states through the pre-selection or post-selection of phase, which means the coherent light source is an advantage over the single-photon source. Among all TF-QKD protocols, the sending or not-sending (SNS) TF-QKD protocol [11] can improve the tolerable threshold of misalignment error in the single-photon interference. Many effects of the SNS protocol in the practical implementation have been investigated to improve its performance [17,18,19,20,21,22,23,24]. In addition, several experiments on the SNS protocol have been performed in the laboratory [25,26] and field [27] to accelerate its application.

Despite the great progress, some deficiencies would cause the security loopholes at the light source in the practical QKD systems. There are three main causes of the security loopholes [28]: the first is the state preparation flaws (SPFs) caused by the finite precision of the modulation devices, the second is the side channels arising from the mode dependencies or due to the Trojan-horse attacks (THAs), and the third is the classical correlations between the sending pulses. Many attempts are devoted to overcome these security loopholes at the source [29,30,31,32,33,34,35,36,37,38] and many experiments have been performed with these methods in different protocols [39,40,41]. Among them, the loss-tolerant (LT) method [30] proposed by Tamaki et al. can tolerant the SPFs at the light source while maintaining a high SKR. The limitation of the LT method is that it requires the emitting states in the qubit space. The limitation is released by the generalized LT (GLT) method [42], which can include SPFs and the effect of side channels without qubit assumption. In addition, the GLT method does not require a detailed characterization of the side channels, which makes it more practical. The last security loophole, i.e., the classical pulses correlations, can be overcome by regarding the leaked information encoded into the correlations of the pulses as side channels [28].

In TF-QKD protocols, one major goal is the long-distance key distribution, which makes Eve could enhance the imperfections of the light sources by exploiting the large channel loss. In this paper, we adopt the SNS protocol to accommodate the SPFs and side channels at the light source. As the SNS protocol eliminates the phase drift with the method of the post-selection, the flaws can be regarded as phase noise and eliminated in this process. The flaws of intensity modulation, e.g., the intensity fluctuations, can also be overcome [24]. To overcome the side channels, we extend the GLT method [42] to the SNS protocol. It may need at least nine actual yields to calculate the transmission rate of the Pauli operators ${\hat{\sigma }}_{t}⊗{\hat{\sigma }}_{t^{\prime }}$ [40]. However, the yields, when Alice and Bob select different bases, cannot be obtained in the SNS protocol. In this paper, according to the characteristic of the states in SNS protocol, we modify the decomposition of the two-mode single-photon states. In this way, only two groups of parameters (two for each group) need to be calculated. To make it more practical, we consider the coherent states as the light sources and analyze how to apply the method in the four-intensity decoy-state SNS protocol.

The paper is arranged as follows. In Section 2, we make a review of the four-intensity decoy-state SNS protocol. We analyze the SNS protocol with flawed and leaky sources in Section 3 and show how to apply the GLT method with the decoy-state method in Section 4. In Section 5, we show the numerical simulations and present the simulation results. Last, the conclusion is given in Section 6.

## 2. Four-Intensity Decoy-State SNS Protocol

In this section, we make a review of the four-intensity decoy-state SNS protocol [11,17].

(1) State preparation. Alice and Bob independently determines the signal and decoy windows with probabilities $p_{z}$ and $p_{x}$. In signal windows, Alice (Bob) prepares the phase-randomized coherent states with intensity $\mu_{z}$ by probability $p_{z1}$ and denotes it as 1 (0), or prepares the vacuum states |0〉 (i.e., not sending) by probability $p_{z0}=1-p_{z1}$ and denotes it as 0 (1). In decoy windows, Alice (Bob) prepares the phase-randomized coherent states $|\sqrt{\mu_{a}}e^{i\theta_{A}}\rangle$, $|\sqrt{\mu_{b}}e^{i\theta_{A}^{\prime }}\rangle$ or |0〉 ($|\sqrt{\mu_{a}}e^{i\theta_{B}}\rangle$, $|\sqrt{\mu_{b}}e^{i\theta_{B}^{\prime }}\rangle$ or |0〉) with probabilities $p_{a}$, $p_{b}$ and $p_{v}=1-p_{a}-p_{b}$, respectively.

(2) Measurement. Charlie performs the interferometric measurements on all twin fields with a beam splitter (BS) and two single-photon detectors (SPDs). The measurement results *d* are announced via public channels, where d=0 or 1 corresponds to only the right or left detector clicks.

(3) Basis announcement and sifting. After repeating the above steps *N* times, Alice and Bob announce their signal and decoy windows through the public channels. Define a *Z* (*X*) window as a time window when both Alice and Bob have determined the signal (decoy) windows. Since the phase of the signal states is unknown to Eve, the signal state is equivalent to a probabilistic mixture of different photon-number states $\sum_{k=0}^{\infty }\frac{e^{-\mu_{z}}\mu_{z}^{k}}{k!}\left|k\rangle \langle k\right|$. Define the $Z_{1}$ windows as a subset of the *Z* windows when only one party determines to send and Alice (Bob) actually sends the single-photon states |1〉. In those *X* windows when they have chosen the same intensity $\mu_{a}$ ($\mu_{b}$), they announce their phase information $\theta_{A}$ and $\theta_{B}$ and denote them as $X_{a}$ ($X_{b}$) windows. Then they sift the effective events, which is defined as one-detector heralded events in *Z* windows and one-detector heralded events with $\theta_{A}$ and $\theta_{B}$ satisfying

(1) $$|\theta_{A}-\theta_{B}-\varphi_{AB}|\leq \Delta /2$$

or

(2) $$|\theta_{A}-\theta_{B}-\pi -\varphi_{AB}|\leq \Delta /2$$

in $X_{a}$ ($X_{b}$) windows, where $\varphi_{AB}$ is set to overcome the phase drift which can be estimated with reference pulses. In this process, they will obtain $n_{t}$ raw bits in the effective *Z* windows, which can be used to distill the secret key bits.

(4) Parameter estimation. They can estimate the bit-flip error rate of the raw bits $E_{z}$ through the error test, the lower bound counting rate ${\underline{s}}_{1}$ and the upper bound phase-flip error rate ${\bar{e}}_{1}^{ph}$ of the single-photon states in $Z_{1}$ windows with the decoy-state method.

(5) Error correction and privacy amplification. Last, they perform error correction on the raw strings and then perform the privacy amplification on the corrected strings.

(6) Key rate formula. With these quantities, the final rate of the secret keys can be expressed as [11,43]

(3) $$R=2p_{z0}\left(1-p_{z0}\right)\mu_{z}e^{-\mu_{z}}{\underline{s}}_{1}\left[1-H\left({\bar{e}}_{1}^{ph}\right)\right]-n_{t}fH\left(E_{z}\right)/N.$$

To improve the performance of the SNS protocol, Alice and Bob can perform the method of actively odd-parity pairing (AOPP) before error correction in the post-processing step [22,23].

## 3. Security Analysis of SNS with Flawed and Leaky Sources

In this section, we analyze how to guarantee the practical security of the SNS protocol with the SPFs and the side channels. In the virtual protocol (described in Appendix A) and the actual protocol, the quantum and classical information available to Eve are the same, which means Eve cannot distinguish and behave differently. Therefore, the security of the actual SNS protocol can be guaranteed once the security of the virtual protocol is proved. In the following, we analyze how to calculate the phase error rate, which quantifies the amount of information leaked to Eve and should be removed in the privacy amplification step. Moreover, the lower bound counting rate of the single-photon states ${\underline{s}}_{1}$ can be estimated with the decoy-state method [17].

The flaws of phase modulation can be overcome directly with the method of the post-selection of phase. In signal windows, Alice and Bob encode bits on their decision to sending or not-sending but not their phase, which makes the signal states immune to the flaws of the phase modulation. However, the phase-randomization assumption should be guaranteed when using the coherent light source. Attacks may be applied when the assumption is violated [44,45]. Fortunately, this assumption can be guaranteed in the SNS protocol once the flaws are known to Alice and Bob. For example, consider the actual phase as $\theta_{A}+\delta \theta_{A}/\pi$ ($\theta_{B}-\delta \theta_{B}/\pi$) for Alice (Bob), where $\theta_{A}$ ($\theta_{B}$) is the expected phase and δ is the deviation of the phase modulation [30,42]. In this scenario, they could ensure $\theta_{A}$ is random to Eve and just regard $\delta \theta_{A}/\pi$ ($\delta \theta_{B}/\pi$) as phase noise, which can be eliminated with post-selection of phase. The flaws of the phase modulation in decoy windows can be overcome in the same way. In the protocol, Alice and Bob could just add $\delta (\theta_{A}+\theta_{B})/\pi$ to $\varphi_{AB}$ and use the modified parameter. Therefore, the flaws of the phase modulation do not affect the security of the SNS protocol and we only consider the side channels in the following.

The side channels may arise from mode dependencies or due to THAs. Mode dependencies mean the optical mode of signal pulses depends on their settings, which means the basis or bit information may be leaked in various degrees, e.g., the polarization, frequency spectrum, and temporal. Moreover, Eve could perform THAs actively by sending strong light into Alice and Bob’s devices and steal information by analyzing the back-reflected light [46,47,48]. On the one hand, we could equip the systems with security patches by adding filters and isolators to resist these THAs. On the other hand, Eve could attack these components, which may compromise the SKR [49]. For these side channels, we overcome it by extending the GLT method [42] to the SNS protocol as discussed in the following.

Now, the analysis focuses on the virtual protocol. In *Z* windows, suppose Alice (Bob) prepares $|\Phi_{z}\rangle_{CE}$ ($|\Phi_{z}\rangle_{C^{\prime }E^{\prime }}$) or vacuum states |0〉 when deciding sending or not-sending, where

(4) $$|\Phi_{z}\rangle_{CE}=a_{z}|\varphi_{z}\rangle_{CE}+b_{z}{|\varphi_{z}^{⊥}\rangle }_{CE},$$

with $|a_{z}{|}^{2}+{\left|b_{z}\right|}^{2}=1$. Here, the subscript *C* and *E* represent Charlie and Eve’s systems, where the latter may include, for example, the trojan light. We assume that $|\varphi_{z}\rangle_{CE}={|1\rangle }_{C}⊗{|0\rangle }_{E}$ is a pure state in the single-mode qubit space, and $|\varphi_{z}^{⊥}\rangle_{CE}$ is orthogonal to $|\varphi_{z}\rangle_{CE}$. Therefore, the system *E* is independent on the system *C* when considering the states $|\varphi_{z}\rangle_{CE}$ and ${|0\rangle }_{CE}$. Ideally, the state prepared by Alice should be $|\Phi_{z}\rangle_{CE}^{ideal}={|\varphi_{z}\rangle }_{CE}$ with no side channels. Please note that the system *C* of the state $|\varphi_{z}^{⊥}\rangle_{CE}$ only include the single-photon component which may have side channels (e.g. with different polarization). The form of the pure state in Equation (4) is the most general independently and identically distributed state. For the mixed states in a single-mode qubit space, the analysis is also applicable by introducing the ancillary systems as discussed in Appendix A. Similarly, suppose $|\Phi_{z}\rangle_{C^{\prime }E^{\prime }}=a_{z}^{\prime }|\varphi_{z}\rangle_{C^{\prime }E^{\prime }}+b_{z}^{\prime }{|\varphi_{z}^{{}^{\prime }⊥}\rangle }_{C^{\prime }E^{\prime }}$ for Bob, where $|\varphi_{z}\rangle_{C^{\prime }E^{\prime }}={|\varphi_{z}\rangle }_{CE}$.

In the following, we assume $|\Phi_{z}\rangle_{C^{\prime }E^{\prime }}={|\Phi_{z}\rangle }_{CE}$ for simplicity. On the one hand, this assumption is not unreasonable in the following analysis. At first glance, this assumption is unreasonable, because, for example, the trojan light of both sides cannot been controlled by Alice and Bob. However, Alice and Bob do not need to characterize the states $|\varphi_{z}^{⊥}\rangle_{CE}$ and $|\varphi_{z}^{{}^{\prime }⊥}\rangle_{C^{\prime }E^{\prime }}$ in the GLT method. Instead, they only need to characterize the lower bound of $a_{z}$ and $a_{z}^{\prime }$, i.e., the amplitude of the state ${|10\rangle }_{CE}$. Therefore, we could replace $a_{z}$ and $a_{z}^{\prime }$ with $\min\{a_{z},a_{z}^{\prime }\}$, and neglect the specific formulas of states $|\varphi_{z}^{⊥}\rangle_{CE}$ and $|\varphi_{z}^{{}^{\prime }⊥}\rangle_{C^{\prime }E^{\prime }}$, which will lead to conservative results. On the other hand, this assumption is not necessary and the analysis can also be applied but will be cumbersome without this assumption.

In the virtual protocol, Alice and Bob prepares the following states in *Z* windows by introducing the local ancillary systems *A* and *B* as

(5) $$|\Psi_{z}^{q}\rangle_{CEC^{\prime }E^{\prime }AB}=\frac{1}{\sqrt{2}}{[|0\Phi_{z}\rangle }_{CEC^{\prime }E^{\prime }}⊗{|01\rangle }_{AB}+{\left(-1\right)}^{q}|\Phi_{z}{0\rangle }_{CEC^{\prime }E^{\prime }}⊗{|10\rangle }_{AB}],$$

where q=0 or 1 corresponds to the phase difference 0 or π between Alice and Bob. In the actual protocol, Alice and Bob only need to prepare $(|0\Phi_{z}\rangle \langle 0\Phi_{z}\left|+\right|\Phi_{z}0\rangle \langle \Phi_{z}0|)/2$ without encoding the phase difference to 0 or π exactly, which makes it immune to the flaws of the phase modulation. To obtain the phase error rate, they could measure the ancillary system *A* and *B* virtually in the basis $\{|\Phi^{0}\rangle ,|\Phi^{1}\rangle \}$ jointly or in the *X* basis separately [11]. Suppose they obtain the bit values $k_{x}$ and $m_{x}$ when measuring the ancillary systems *A* and *B* in the *X* basis. Depending on the phase difference *q* and the measurement result *d*, the phase error rate can be defined as

(6) $$E_{\left(q,d\right)}=\frac{\sum_{k,m\in \{0,1\},k⨁m=q⨁d}Y_{k_{x},m_{x}}^{(q,d),vir}}{\sum_{k,m\in \{0,1\}}Y_{k_{x},m_{x}}^{(q,d),vir}},$$

where $Y_{k_{x},m_{x}}^{(q,d),vir}$ is the conditional probability that Alice and Bob obtain bit value $k_{x}$ and $m_{x}$ in the *X* basis and Charlie announces the measurement result *d* conditioned on that they prepare the states $|\Psi_{z}^{q}\rangle_{CEC^{\prime }E^{\prime }AB}$. In fact, the states sent out by Alice and Bob are the same for different *q*, which can be seen by taking partial trace over the systems *A* and *B*. In particular, we have $Y_{k_{x},m_{x}}^{(q,d),vir}=Y_{k_{x}^{\prime },m_{x}^{\prime }}^{(\bar{q},d),vir}$ when $k_{x}+m_{x}=k_{x}^{\prime }+m_{x}^{\prime }+1\left(\mathrm{mod}\;2\right)$. Hence, the phase error rate corresponding to the measurement result *d* can be defined as

(7) $$E_{d}=\frac{\sum_{k,m,q\in \{0,1\},k⨁m=q⨁d}Y_{k_{x},m_{x}}^{(q,d),vir}}{\sum_{k,m,q\in \{0,1\}}Y_{k_{x},m_{x}}^{(q,d),vir}}.$$

With these phase error rates, the cost of performing privacy amplification to remove the correlations between the sifted key bits and Eve is $\left[n_{0}H\left(E_{0}\right)+n_{1}H\left(E_{1}\right)\right]/\left(n_{0}+n_{1}\right)$, where $n_{d}$ is the number of the effective events corresponds to *d* in *Z* windows. We can define the overall phase error rate as

(8) $$E_{ph}=\frac{E_{0}n_{0}+E_{1}n_{1}}{n_{0}+n_{1}}.$$

It will consume more bits to use the formula $E_{ph}$ than $E_{d}$ when $n_{0}\neq n_{1}$, which may be caused by the imperfect beam splitting ratio of the beam splitter (BS) or the mismatch of the detection efficiency of the two SPDs on Charlie’s side. Therefore, we consider the formulas in Equation (7) in this paper.

To obtain the denominator of $E_{d}$ in Equation (7), we define $Y_{k_{z},m_{z}}^{\left(q,d\right)}$ as the conditional probability that Alice and Bob obtains bit values $k_{z}$ and $m_{z}$ when measuring the ancillary systems *A* and *B* in the *Z* basis and only the *d* detector clicks on Charlie’s side conditioned on that Alice and Bob prepare the states $|\Psi_{z}^{q}\rangle_{CEC^{\prime }E^{\prime }AB}$. Please note that Alice and Bob’s measurements on the ancillary systems *A* and *B* in the *Z* or *X* basis is virtual, which can be regarded as ideal measurements with no difference in detection efficiency. Therefore, the denominator of $E_{d}$ is equal to $\sum_{k,m,q\in \{0,1\}}Y_{k_{z},k_{z}}^{\left(q,d\right)}$, which can be observed in the experiment directly. This means we only need to calculate the $Y_{k_{x},m_{x}}^{(q,d),vir}$ in the numerator of $E_{d}$.

In the virtual protocol, after obatining the bit values $k_{x}$ and $m_{x}$ in the *X* basis, they will send Charlie the (unnormalized) state

(9) $${\hat{\sigma }}_{CEC^{\prime }E^{\prime },k_{x},m_{x}}^{q,vir}=\mathrm{Tr}(I_{CEC^{\prime }E^{\prime }}⊗|k_{x}\rangle \langle k_{x}{|}_{A}⊗|m_{x}\rangle \langle m_{x}{|}_{B}|\Psi_{z}^{q}\rangle \langle \Psi_{z}^{q}{|}_{CEC^{\prime }E^{\prime }AB}).$$

Substituting Equation (5) into Equation (9), we obtain ${\hat{\sigma }}_{CEC^{\prime }E^{\prime },k_{x},m_{x}}^{q,vir}=|\varphi_{k_{x},m_{x}}^{q,vir}\rangle \langle \varphi_{k_{x},m_{x}}^{q,vir}{|}_{CEC^{\prime }E^{\prime }}$ with

(10) $$|\varphi_{k_{x},m_{x}}^{q,vir}\rangle_{CEC^{\prime }E^{\prime }}=\frac{a_{z}}{2}|\gamma_{k_{x},m_{x}}^{q,vir}\rangle_{CEC^{\prime }E^{\prime }}+\frac{b_{z}}{2}{|\gamma_{k_{x},m_{x}}^{⊥,q,vir}\rangle }_{CEC^{\prime }E^{\prime }}.$$

Here, the normalized state $|\varphi_{k_{x},m_{x}}^{q,vir}\rangle_{CEC^{\prime }E^{\prime }}$ is

(11) $$|\gamma_{k_{x},m_{x}}^{q,vir}\rangle_{CEC^{\prime }E^{\prime }}=\frac{1}{\sqrt{2}}{[|0\varphi_{z}\rangle }_{CEC^{\prime }E^{\prime }}+{\left(-1\right)}^{q+k+m}|\varphi_{z}0\rangle_{CEC^{\prime }E^{\prime }}].$$

Since the inner products ${\langle \varphi_{z}|0\rangle }_{CE}$ and ${\langle \varphi_{z}^{⊥}|0\rangle }_{CE}$ are equal to 0, the normalized states $|\gamma_{k_{x},m_{x}}^{⊥,q,vir}\rangle_{CEC^{\prime }E^{\prime }}$ that is orthogonal to $|\varphi_{k_{x},m_{x}}^{q,vir}\rangle_{CEC^{\prime }E^{\prime }}$ can be shown as

(12) $$|\gamma_{k_{x},m_{x}}^{⊥,q,vir}\rangle_{CEC^{\prime }E^{\prime }}=\frac{1}{\sqrt{2}}{[|0\varphi_{z}^{⊥}\rangle }_{CEC^{\prime }E^{\prime }}+{\left(-1\right)}^{q+k+m}|\varphi_{z}^{⊥}0\rangle_{CEC^{\prime }E^{\prime }}].$$

Therefore, the conditional probability $Y_{k_{x},m_{x}}^{(q,d),vir}$ can be expressed as

(13) $$Y_{k_{x},m_{x}}^{(q,d),vir}=\mathrm{Tr}\left({\hat{D}}_{d}{\hat{\sigma }}_{CEC^{\prime }E^{\prime },k_{x},m_{x}}^{q,vir}\right),$$

where ${\hat{D}}_{d}=\sum_{k}{\hat{A}}_{k}^{†}{\hat{M}}_{d}{\hat{A}}_{k}$ corresponds to Eve’s action represented by the Kraus operators $\left\{{\hat{A}}_{k}\right\}$ and Charlie’s measurement with POVMs $\left\{{\hat{M}}_{d}\right\}$. Here, we assume that Eve applies the same quantum operation to every pulses, which corresponds to the collective attack. The analysis can be extended to coherent attacks which is discussed in Appendix B. Combining Equations (9), (10) and (13), we obtain the following expression

(14) $$\begin{array}{cc} Y_{k_{x},m_{x}}^{(q,d),vir}= & \frac{1}{4}[|a_{z}{|}^{2}\mathrm{Tr}({\hat{D}}_{d}|\gamma_{k_{x},m_{x}}^{q,vir}\rangle \langle \gamma_{k_{x},m_{x}}^{q,vir}{|}_{CEC^{\prime }E^{\prime }})+\mathrm{Tr}({\hat{D}}_{d}(a_{z}^{*}b_{z}|\gamma_{k_{x},m_{x}}^{⊥,q,vir}\rangle \langle \gamma_{k_{x},m_{x}}^{q,vir}{|}_{CEC^{\prime }E^{\prime }}\\ \; & +a_{z}b_{z}^{*}|\gamma_{k_{x},m_{x}}^{q,vir}\rangle \langle \gamma_{k_{x},m_{x}}^{⊥,q,vir}{|}_{CEC^{\prime }E^{\prime }}+|b_{z}{|}^{2}|\gamma_{k_{x},m_{x}}^{⊥,q,vir}\rangle \langle \gamma_{k_{x},m_{x}}^{⊥,q,vir}{|}_{CEC^{\prime }E^{\prime }}))]. \end{array}$$

The lower and upper bounds of $Y_{k_{x},m_{x}}^{(q,d),vir}$ can be obtained by calculating the bounds of the first and second terms in Equation (14), separately.

The density matrix of the state $|\gamma_{k_{x},m_{x}}^{q,vir}\rangle_{CEC^{\prime }E^{\prime }}$ can be decomposed as

(15) $$|\gamma_{k_{x},m_{x}}^{q,vir}\rangle \langle \gamma_{k_{x},m_{x}}^{q,vir}{|}_{CEC^{\prime }E^{\prime }}=\left[\rho_{x}+{\left(-1\right)}^{q+k+m}\rho_{y}\right]⊗{\left|00\rangle \langle 00\right|}_{EE^{\prime }},$$

with $\rho_{x}={(|01\rangle \langle 01|}_{CC^{\prime }}+|10\rangle \langle 10{|}_{CC^{\prime }})/2$ and $\rho_{y}={(|01\rangle \langle 10|}_{CC^{\prime }}+|10\rangle \langle 01{|}_{CC^{\prime }})/2$. Here, $\rho_{x}+{\left(-1\right)}^{q+k+m}\rho_{y}$ is the density operator of the two-mode single-photon state $(|01\rangle +{\left(-1\right)}^{q+k+m}\left|10\rangle \right)/\sqrt{2}$, but $\rho_{x}$ and $\rho_{y}$ are not density operator of some particular states, which is different from the original GLT method [42]. Therefore, the component in the first term of Equation (14) can be expressed as

(16) $$\mathrm{Tr}({\hat{D}}_{d}|\gamma_{k_{x},m_{x}}^{q,vir}\rangle \langle \gamma_{k_{x},m_{x}}^{q,vir}{|}_{CEC^{\prime }E^{\prime }})=x_{d}+{\left(-1\right)}^{q+k+m}y_{d},$$

where $x_{d}=\mathrm{Tr}\left[{\hat{D}}_{d}\rho_{x}\right]$ and $y_{d}=\mathrm{Tr}\left[{\hat{D}}_{d}\rho_{y}\right]$. Here, $x_{d}+{\left(-1\right)}^{q+k+m}y_{d}$ can be regarded as the transmission rate of the state $(|01\rangle +{\left(-1\right)}^{q+k+m}\left|10\rangle \right)/\sqrt{2}$. In this way, we only need to calculate the parameters $x_{d}$ and $y_{d}$. The number of parameters that need to be solved is reduced. The second term of Equation (14) can be written as $\mathrm{Tr}({\hat{D}}_{d}N_{z})$, where the matrix

(17) $$N_{z}=\left[\begin{array}{ccc} |b_{z}{|}^{2} & a_{z}^{*}b_{z} & 0_{1\times 2}\\ a_{z}b_{z}^{*} & 0_{1\times 1} & 0_{1\times 2}\\ 0_{2\times 1} & 0_{2\times 1} & 0_{2\times 2} \end{array}\right].$$

with eigenvalues

(18) $$\begin{array}{c} \lambda_{\min}^{z}=\frac{|b_{z}{|}^{2}-\left|b_{z}\right|\sqrt{|b_{z}{|}^{2}+4{\left|a_{z}\right|}^{2}}}{2},\\ \lambda_{\max}^{z}=\frac{|b_{z}{|}^{2}+\left|b_{z}\right|\sqrt{|b_{z}{|}^{2}+4{\left|a_{z}\right|}^{2}}}{2}, \end{array}$$

and $\lambda_{0}=0$. Using the properties of POVMs, the eigenvalues of ${\hat{D}}_{d}$ are between 0 and 1. Hence, $\mathrm{Tr}({\hat{D}}_{d}N_{z})$ can be bounded by $\lambda_{\min}^{z}\leq \mathrm{Tr}\left({\hat{D}}_{d}N_{z}\right)\leq \lambda_{\max}^{z}$.

In the following, we analyze how to obtain the bounds of $x_{d}$, $y_{d}$ with the events in *X* windows. Consider the $X_{\alpha }$ windows in the virtual protocol and assume they prepare the states as

(19) $$|\Psi_{\alpha }^{\left(\theta_{A},\theta_{B}\right)}\rangle_{CEC^{\prime }E^{\prime }AB}=\frac{1}{\sqrt{2}}{[e^{i\theta_{B}}|0\Phi_{\alpha }\rangle }_{CEC^{\prime }E^{\prime }}⊗{|01\rangle }_{AB}+e^{i\theta_{A}}|\Phi_{\alpha }{0\rangle }_{CEC^{\prime }E^{\prime }}⊗{|10\rangle }_{AB}],$$

where

(20) $$|\Phi_{\alpha }\rangle_{CE}=a_{\alpha }|\varphi_{\alpha }\rangle_{CE}+b_{\alpha }{|\varphi_{\alpha }^{⊥}\rangle }_{CE}.$$

Similar to *Z* windows, we suppose that $|\varphi_{\alpha }\rangle_{CE}={|1\rangle }_{C}⊗{|0\rangle }_{E}$ is a pure state in a single-mode qubit space and $|\varphi_{\alpha }^{⊥}\rangle_{CE}$ is orthogonal to $|\varphi_{\alpha }\rangle_{CE}$. Therefore, the system *E* is independent on the system *C* for the states $|\varphi_{\alpha }\rangle_{CE}$, $|\varphi_{z}\rangle_{CE}$, and ${|00\rangle }_{CE}$, which means the side channels are only included in the states $|\varphi_{\alpha }^{⊥}\rangle_{CE}$ and $|\varphi_{z}^{⊥}\rangle_{CE}$.

Define the conditional probability $Y_{k_{x},m_{x}}^{(c,d),\alpha ,\Delta }$ that Alice and Bob obatin $k_{x}$ and $m_{x}$ in the *X* basis and only the *d* detector clicks on Charlie’s side conditioned on that Alice and Bob prepare the state $|\Psi_{\alpha }^{\left(\theta_{A},\theta_{B}\right)}\rangle_{CEC^{\prime }E^{\prime }AB}$ with phase $\theta_{A}$ and $\theta_{B}$ satisfying Equation (1) or Equation (2) (denoted as c=0 or 1, respectively). After measuring the ancillary systems *A* and *B* in the *X* basis, they will sent Charlie the (unnormalized) state

(21) $${\hat{\sigma }}_{CEC^{\prime }E^{\prime },k_{x},m_{x}}^{(\theta_{A},\theta_{B}),\alpha }=\mathrm{Tr}(I_{CEC^{\prime }E^{\prime }}⊗|k_{x}\rangle \langle k_{x}{|}_{A}⊗|m_{x}\rangle \langle m_{x}{|}_{B}|\Psi_{\alpha }^{\left(\theta_{A},\theta_{B}\right)}\rangle \langle \Psi_{\alpha }^{\left(\theta_{A},\theta_{B}\right)}{|}_{CEC^{\prime }E^{\prime }AB}).$$

Substituting Equation (19) into Equation (21), we obtain ${\hat{\sigma }}_{CEC^{\prime }E^{\prime },k_{x},m_{x}}^{(\theta_{A},\theta_{B}),\alpha }=|\varphi_{k_{x},m_{x}}^{(\theta_{A},\theta_{B}),\alpha }\rangle$$\langle \varphi_{k_{x},m_{x}}^{(\theta_{A},\theta_{B}),\alpha }{|}_{CEC^{\prime }E^{\prime }}$ with the unnormalized state

(22) $$|\varphi_{k_{x},m_{x}}^{(\theta_{A},\theta_{B}),\alpha }\rangle_{CEC^{\prime }E^{\prime }}=\frac{a_{\alpha }}{2}|\gamma_{k_{x},m_{x}}^{(\theta_{A},\theta_{B}),\alpha }\rangle_{CEC^{\prime }E^{\prime }}+\frac{b_{\alpha }}{2}{|\gamma_{k_{x},m_{x}}^{⊥,(\theta_{A},\theta_{B}),\alpha }\rangle }_{CEC^{\prime }E^{\prime }}.$$

The normalized state $|\gamma_{k_{x},m_{x}}^{(\theta_{A},\theta_{B}),\alpha }\rangle_{CEC^{\prime }E^{\prime }}$ can be shown as

(23) $$|\gamma_{k_{x},m_{x}}^{(\theta_{A},\theta_{B}),\alpha }\rangle_{CEC^{\prime }E^{\prime }}=\frac{1}{\sqrt{2}}{[e^{i\theta_{B}}|0\varphi_{\alpha }\rangle }_{CEC^{\prime }E^{\prime }}+{\left(-1\right)}^{k+m}e^{i\theta_{A}}|\varphi_{\alpha }0\rangle_{CEC^{\prime }E^{\prime }}].$$

And the normalized state $|\gamma_{k_{x},m_{x}}^{⊥,(\theta_{A},\theta_{B}),\alpha }\rangle_{CEC^{\prime }E^{\prime }}$ which is orthogonal to $|\gamma_{k_{x},m_{x}}^{(\theta_{A},\theta_{B}),\alpha }\rangle_{CEC^{\prime }E^{\prime }}$ can be shown as

(24) $$|\gamma_{k_{x},m_{x}}^{⊥,(\theta_{A},\theta_{B}),\alpha }\rangle_{CEC^{\prime }E^{\prime }}=\frac{1}{\sqrt{2}}{[e^{i\theta_{B}}|0\varphi_{\alpha }^{⊥}\rangle }_{CEC^{\prime }E^{\prime }}+{\left(-1\right)}^{k+m}e^{i\theta_{A}}|\varphi_{\alpha }^{⊥}0\rangle_{CEC^{\prime }E^{\prime }}].$$

Therefore, the conditional probability $Y_{k_{x},m_{x}}^{(c,d),\alpha ,\Delta }$ can be expressed as

(25) $$\begin{array}{c} Y_{k_{x},m_{x}}^{(c,d),\alpha ,\Delta }=\frac{1}{\Delta }\int_{\pi \delta (c-1)-\Delta /2}^{\pi \delta (c-1)+\Delta /2}\mathrm{Tr}\left({\hat{D}}_{d}{\hat{\sigma }}_{CEC^{\prime }E^{\prime },k_{x},m_{x}}^{\left(\theta_{A},\theta_{B}\right)}\right)d\left(\theta_{A}-\theta_{B}\right)\\ =\frac{1}{4}\left[\right|a_{\alpha }{|}^{2}\left(x_{d}+2{\left(-1\right)}^{\left(c+k+m\right)}\left(y_{d}/\Delta \right)\sin\left(\Delta /2\right)\right)+\mathrm{Tr}\left({\hat{D}}_{d}N_{\alpha }\right)], \end{array}$$

where δ(x)=1 when x=0, and δ(x)=0 when x≠0. The matrix $N_{\alpha }$ can be shown as

(26) $$N_{\alpha }=\left[\begin{array}{ccc} |b_{\alpha }{|}^{2} & a_{\alpha }^{*}b_{\alpha } & 0_{1\times 2}\\ a_{\alpha }b_{\alpha }^{*} & 0_{1\times 1} & 0_{1\times 2}\\ 0_{2\times 1} & 0_{2\times 1} & 0_{2\times 2} \end{array}\right].$$

Therefore, $\mathrm{Tr}({\hat{D}}_{d}N_{\alpha })$ can be bounded as $\lambda_{\min}^{\alpha }\leq \mathrm{Tr}\left({\hat{D}}_{d}N_{\alpha }\right)\leq \lambda_{\max}^{\alpha }$, where

(27) $$\begin{array}{c} \lambda_{\min}^{\alpha }=\frac{|b_{\alpha }{|}^{2}-\left|b_{\alpha }\right|\sqrt{|b_{\alpha }{|}^{2}+4{\left|a_{\alpha }\right|}^{2}}}{2},\\ \lambda_{\max}^{\alpha }=\frac{|b_{\alpha }{|}^{2}+\left|b_{\alpha }\right|\sqrt{|b_{\alpha }{|}^{2}+4{\left|a_{\alpha }\right|}^{2}}}{2}, \end{array}$$

are the eigenvalues of the matrix $N_{\alpha }$.

Set $\Delta_{2}$ be equal to the parameter Δ selected in the protocol in Equations (1) and (2), and $\Delta_{1}=2\Delta$, we obtain two linear equalities of $Y_{k_{x},m_{x}}^{\left(c,d\right),\alpha ,\Delta_{1}}$ and $Y_{k_{x},m_{x}}^{\left(c,d\right),\alpha ,\Delta_{2}}$ according to Equation (25). By solving the linear equalities, we obtain

(28) $$x_{d}=\frac{4}{|a_{\alpha }{|}^{2}}\frac{\Delta_{1}Y_{k_{x},m_{x}}^{\left(c,d\right),\alpha ,\Delta_{1}}\sin\left(\Delta_{2}/2\right)-\Delta_{2}Y_{k_{x},m_{x}}^{\left(c,d\right),\alpha ,\Delta_{2}}\sin\left(\Delta_{1}/2\right)}{\Delta_{1}\sin\left(\Delta_{2}/2\right)-\Delta_{2}\sin\left(\Delta_{1}/2\right)}-\frac{\mathrm{Tr}({\hat{D}}_{d}N_{\alpha })}{|a_{\alpha }{|}^{2}},$$

and

(29) $$y_{d}=\frac{2\Delta_{1}\Delta_{2}}{{\left(-1\right)}^{c+k+m}{\left|a_{\alpha }\right|}^{2}}\frac{Y_{k_{x},m_{x}}^{\left(c,d\right),\alpha ,\Delta_{2}}-Y_{k_{x},m_{x}}^{\left(c,d\right),\alpha ,\Delta_{1}}}{\Delta_{1}\sin\left(\Delta_{2}/2\right)-\Delta_{2}\sin\left(\Delta_{1}/2\right)}.$$

Therefore, $x_{d}$ and $y_{d}$ can be obtained with the yields $Y_{k_{x},m_{x}}^{(c,d),\alpha ,\Delta }$. In the actual protocol, we analyze how to obtain the yields $Y_{k_{x},m_{x}}^{(c,d),\alpha ,\Delta }$ with the decoy-state method in the next section.

## 4. Parameter Estimation

In the actual protocol, Alice and Bob do not have the single-photon source and will prepare the coherent states. They neither prepare the ancillary systems *A* and *B* nor perform the local measurement. Therefore, we need to reduce it to the actual protocol and analyze how to calculate the yields $Y_{k_{x},m_{x}}^{(c,d),\alpha ,\Delta }$ in the actual protocol.

In the virtual protocol, consider that Alice and Bob measure the ancillary systems *A* and *B* in the basis $\{|\Phi^{0}\rangle ,|\Phi^{1}\rangle \}$ jointly, we define $Y_{s}^{(c,d),\alpha ,\Delta }=\sum_{k_{x},m_{x},k_{x}⨁m_{x}=s}Y_{k_{x},m_{x}}^{(c,d),\alpha ,\Delta }$. Therefore we have $Y_{k_{x},m_{x}}^{(c,d),\alpha ,\Delta }=Y_{k_{x}⨁m_{x}}^{(c,d),\alpha ,\Delta }/2$. Actually, the states sent are the same for two different *s*, which means that $Y_{s}^{(c,d),\alpha ,\Delta }=Y_{\bar{s}}^{(c,d),\alpha ,\Delta }$. When the parameter *c* is announced, the parameter $Y_{s}^{(c,d),\alpha ,\Delta }$ can be obtained without knowing the parameter *s*. In this way, the virtual measurements on the ancillary systems *A* and *B* can be eliminated by regarding the measurement results as *s*. In the following, we analyze how to obtain the parameter $Y_{s}^{(c,d),\alpha ,\Delta }$ with the four-intensity decoy-state method.

In the actual protocol, the ideal two-mode weak coherent states in $X_{\alpha }$ windows prepared by them are $|\sqrt{\mu_{\alpha }}e^{i\theta_{A}}\rangle ⊗|\sqrt{\mu_{\alpha }}e^{i\theta_{B}}\rangle$ (α∈{a,b}) with the restriction of Equations (1) and (2). By introducing two independent variables $\delta_{\pm }=\left(\theta_{A}\pm \theta_{B}\right)/2$, we can integrate the two-mode state on variable $\delta_{+}$ and obtain a classical mixture

(30) $$\sum_{t=0}^{\infty }p_{t}^{\alpha }|\psi_{t}\rangle \langle \psi_{t}|,$$

with $|\psi_{t}\rangle$ being the state of total photon number *t* for the two-mode state and $p_{t}^{\alpha }$ being its probability. To be specific,

(31) $$|\psi_{t}\rangle =\frac{1}{\sqrt{2^{n}}}\sum_{j=0}^{t}\sqrt{C_{t}^{j}}e^{i[j\theta_{A}+\left(t-j\right)\theta_{B}]}|j,t-j\rangle ,$$

with probability

(32) $$p_{t}^{\alpha }=e^{-2\mu_{\alpha }}\frac{2^{t}\mu_{a}^{t}}{t!}.$$

Define $Q_{\alpha }^{(c,d),\Delta }$ as the conditional probability that the measurement result is *d* conditioned on that they both send the coherent states with intensity $\mu_{\alpha }$, the phase slice is Δ, and their phases satisfy Equation (1) (c=0) or Equation (2) (c=1). Here, $Q_{\alpha }^{(c,d),\Delta }$ can be obtained by statistics in the actual protocol. According to Equation (30), we have

(33) $$Q_{\alpha }^{(c,d),\Delta }=\sum_{t=0}^{\infty }p_{t}^{\alpha }Y_{\left(t\right)}^{(c,d),\Delta },$$

where $Y_{\left(t\right)}^{(c,d),\Delta }$ is the probability that the measurement result is *d* when the two-mode state is $|\psi_{t}\rangle$ with phases satisfying *c* and Δ. Thus, we have $Y_{s}^{(d),\alpha ,\Delta }=Y_{\left(1\right)}^{(c,d),\Delta }/2$.

In this paper, we consider the four-intensity decoy states and the value of $Y_{\left(1\right)}^{(c,d),\Delta }$ cannot be calculated precisely. However, we can obtain the bounds of $Y_{\left(1\right)}^{(c,d),\Delta }$ analytically or by linear programming. We give an example, which is used in the simulation in Section 5, as follows.

Using two decoy states, i.e., $Q_{a}^{(c,d),\Delta }$ and $Q_{b}^{(c,d),\Delta }$, a crude upper bound of $Y_{\left(1\right)}^{(c,d),\Delta }$ can be given by abandoning the parts when t≥2 as

(34) $$Q_{\alpha }^{(c,d),\Delta }=\sum_{t=0}^{\infty }p_{t}^{\alpha }Y_{\left(t\right)}^{(c,d),\Delta }\geq p_{0}^{\alpha }Y_{0}+p_{1}^{\alpha }Y_{\left(1\right)}^{(c,d),\Delta },$$

where $Y_{0}$ can be estimated with the events when they both send the vacuum states in *X* windows. Hence, the upper bound of $Y_{\left(1\right)}^{(c,d),\Delta }$ can be expressed by

(35) $$Y_{\left(1\right)}^{(c,d),\Delta }\leq \frac{Q_{\alpha }^{(c,d),\Delta }-p_{0}^{\alpha }Y_{0}}{p_{1}^{\alpha }}.$$

For the lower bound of $Y_{\left(1\right)}^{(c,d),\Delta }$, we consider the linear combination as

(36) $$\begin{array}{cc} \; & p_{2}^{b}Q_{a}^{(c,d),\Delta }-p_{2}^{a}Q_{b}^{(c,d),\Delta }-p_{0}^{a}p_{2}^{b}Y_{0}+p_{2}^{a}p_{0}^{b}Y_{0}\\ = & p_{1}^{a}p_{2}^{b}Y_{\left(1\right)}^{(c,d),\Delta }-p_{2}^{a}p_{1}^{b}Y_{\left(1\right)}^{(c,d),\Delta }+\sum_{t=3}^{\infty }\left(p_{t}^{a}p_{2}^{b}-p_{2}^{a}p_{t}^{b}\right)Y_{\left(t\right)}^{(c,d),\Delta }. \end{array}$$

The last item of Equation (36) satisfies

(37) $$\sum_{t=3}^{\infty }\left(p_{t}^{a}p_{2}^{b}-p_{2}^{a}p_{t}^{b}\right)Y_{\left(t\right)}^{(c,d),\Delta }=e^{-2(\mu_{a}+\mu_{b})}\mu_{a}^{2}\mu_{b}^{2}\sum_{t=3}^{\infty }\frac{2^{t+1}}{t!}\left(\mu_{a}^{t-2}-\mu_{b}^{t-2}\right)Y_{\left(t\right)}^{(c,d),\Delta }\leq 0.$$

Hence, rewrite Equation (36) with Equation (37), the lower bound of $Y_{\left(1\right)}^{(c,d),\Delta }$ is given by

(38) $$Y_{\left(1\right)}^{(c,d),\Delta }\geq \frac{p_{2}^{b}Q_{a}^{(c,d),\Delta }-p_{2}^{a}Q_{b}^{(c,d),\Delta }+\left(p_{2}^{a}p_{0}^{b}-p_{0}^{a}p_{2}^{b}\right)Y_{0}}{4e^{-2(\mu_{a}+\mu_{b})}\mu_{a}\mu_{b}\left(\mu_{b}-\mu_{a}\right)}.$$

Last, we note that $0\leq Y_{\left(1\right)}^{(c,d),\Delta }\leq 1$.

In this way, the upper bound of the phase error rate can be estimated. We have released the restriction of single-photon states and the preparation of ancillary systems. The above formulas can be used directly in the actual protocol with the decoy-state method.

## 5. Simulation

In this section, we simulate the performance of the four-intensity decoy-state SNS protocol with AOPP with a particular leaky source. For this purpose, we only consider the polarization side channels and a particular THA.

Suppose the state of system *C* ($C^{\prime }$) is dependent on the polarization which can be decomposed as

(39) $$|\Omega_{\beta }\rangle_{C}=\cos\theta_{\beta }|\omega_{\beta }\rangle_{HC}+\sin\theta_{\beta }{|\omega_{\beta }^{⊥}\rangle }_{VC},$$

where β∈{z,a,b}, and the subscripts *H* and *V* represent the horizontal and vertical polarization modes. And for the THA, consider the trojan light as the system *E* ($E^{\prime }$) shown as

(40) $$|\xi_{\beta }\rangle_{E}=C_{I\beta }{|e\rangle }_{E}+C_{D\beta }{|e_{\beta }\rangle }_{E},$$

with $C_{I\beta }^{2}+C_{D\beta }^{2}=1$. Here, the state ${|e\rangle }_{E}$ is independent on Alice and Bob’s choice while $|e_{\beta }\rangle_{E}$ is not. Then Equations (4) and (20) can be expressed as

(41) $$\begin{array}{cc} |\Phi_{\beta }\rangle_{CE}= & \cos\theta_{\beta }C_{I\beta }|\omega_{\beta }\rangle_{HC}{|e\rangle }_{E}+\cos\theta_{\beta }C_{D\beta }|\omega_{\beta }\rangle_{HC}{|e_{\beta }\rangle }_{E}\\ \; & +\sin\theta_{\beta }|\omega_{\beta }^{⊥}\rangle_{VC}⊗{(C_{I\beta }|e\rangle }_{E}+C_{D\beta }|e_{\beta }\rangle_{E}), \end{array}$$

where the first item corresponds to $a_{\beta }{|\varphi_{\beta }\rangle }_{CE}$ while others correspond to $b_{\beta }{|\varphi_{\beta }^{⊥}\rangle }_{CE}$. If we regard the states $|\xi_{\beta }\rangle_{E}$ as a coherent state with intensity $\nu_{\beta }$, we have ${|e\rangle }_{E}=|0\rangle$ with $C_{I\beta }=e^{-\nu_{\beta }/2}$. The effects of the actual systems on the parameters $\theta_{\beta }$ and $\nu_{\beta }$ may be complicated, which makes it diffcult to be charactered. Fortunately, the GLT method can be applied provided the upper bounds of $\theta_{\beta }$ and $\nu_{\beta }$ denoted as $\theta =\max_{\beta }\left\{\theta_{\beta }\right\}$ and $\nu =\max_{\beta }\left\{\nu_{\beta }\right\}$. Therefore, we consider the parameters θ and ν as the upper bounds of side channels for both signal and decoy states in the simulation. In this way, we can simplify the numerical simulation with only two parameters θ and ν but not six parameters $\theta_{\beta }$ and $\nu_{\beta }$ (β∈{z,a,b}). In practical QKD systems, the parameters θ can be charactered using quantum state tomography [40], and the parameters ν can be charactered by monitoring the intensity of the trojan light before fixed attenuation or analyzing its possible maximum according to the isolation of reverse and forward.

We simulate the observed values with the experimental parameters about the actual devices in Table 1, and the parameters about the intensities and probabilities in different windows in Table 2 [26].

**Figure 1:**
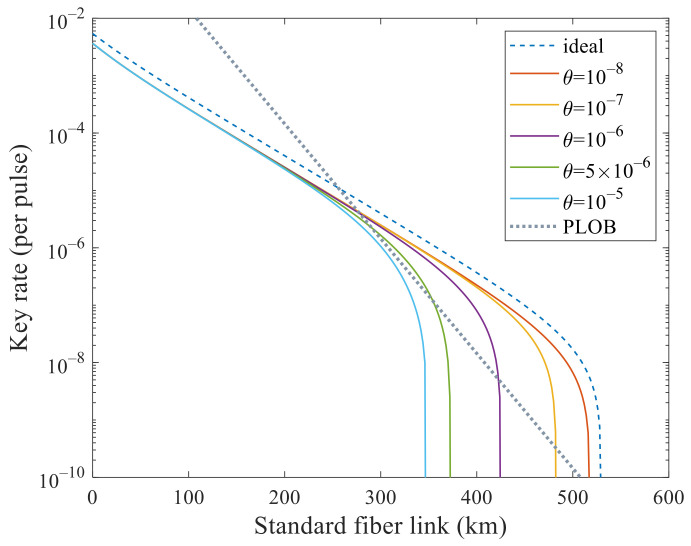
Secret key rate (per pulse) in logarithmic scale versus transmission distance between Alice and Bob when only one kind of side channel is considered. The dashed line corresponds to the ideal case without side channels that is estimated with the method in [11,17,22]. In addition, the dotted line is the PLOB bound. The solid lines correspond to the cases with different θ when ν=0 estimated with the method in this paper. The lines correspond to θ=0 and $10^{-8}$ are superimposed, which means that it will not affect SKR when θ is less than $10^{-8}$. It can beat the PLOB bound when θ increases to $5\times 10^{-6}$.

**Figure 2:**
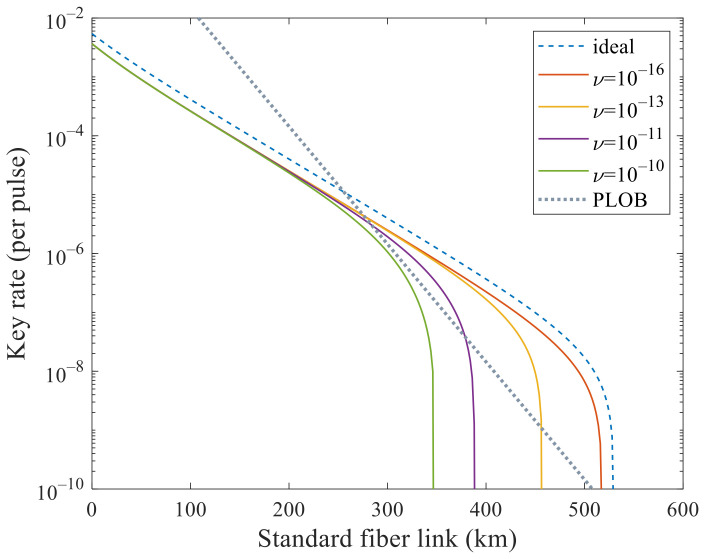
Secret key rate (per pulse) in logarithmic scale versus transmission distance between Alice and Bob when only one kind of side channel is considered. The dashed line corresponds to the ideal case without side channels that is estimated with the method in [11,17,22]. In addition, the dotted line is the PLOB bound. The solid lines correspond to the cases with different ν when θ=0 estimated with the method in this paper. The solid lines correspond to ν=0 and $10^{-16}$ are superimposed, which means that it will not affect SKR when ν is less than $10^{-16}$. It can surpass the PLOB bound when increasing ν to around $10^{-11}$.

**Figure 3:**
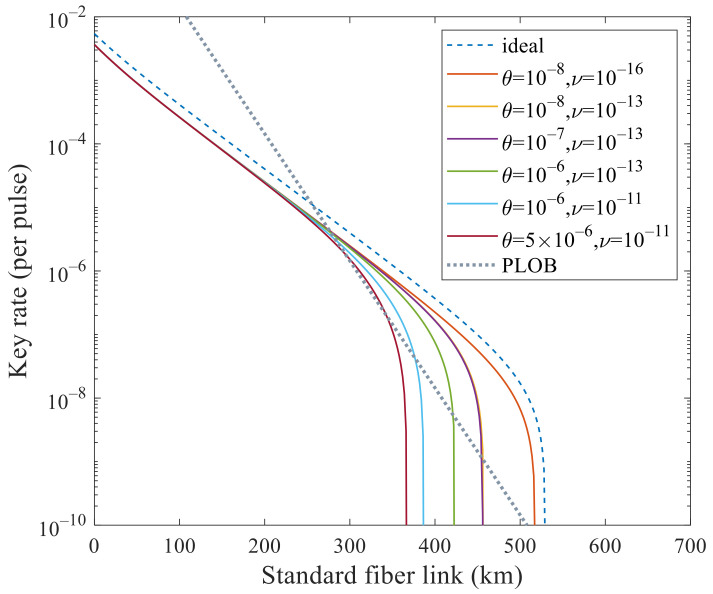
Secret key rate (per pulse) in logarithmic scale versus transmission distance between Alice and Bob with both side channels θ and ν. The dashed line corresponds to the ideal case without side channels that is estimated with the method in [11,17,22]. In addition, the dotted line is the PLOB bound. The SKR when θ=0 and ν=0 overlaps with that when $\theta =10^{-8}$ and $\nu =10^{-16}$. The SKR when $\theta =10^{-8}$, $\nu =10^{-13}$ and $\theta =10^{-7}$, $\nu =10^{-13}$ are superimposed. In addition, the SKR with $\theta =10^{-6}$ and $\nu =10^{-11}$ surpass the PLOB bound at 296 to 340 km.

We mainly analyze its performance to surpass PLOB bound [9] under these experimental parameters with flawed and leaky sources. In Figure 1 and Figure 2, we analyze the effects of these two kinds of side channels separately. In Figure 1, the SKR can beat the PLOB bound when θ increases to $5\times 10^{-6}$ but cannot when $10^{-5}$. In Figure 2, the SKR can beat the PLOB bound when ν increases to $10^{-11}$ but cannot when $10^{-10}$. Combining these two values, i.e., $\theta =5\times 10^{-6}$ and $\nu =10^{-11}$, we can see that the SKR still can surpass the PLOB bound at 296 to 340 km in Figure 3. When $\theta =10^{-8}$ and $\nu =10^{-16}$, the SKR overlaps with that when θ=0 and ν=0, which means that the side channels will not affect the SKR at this time. We can see that the SKR is hardly affected by the side channels at a short distance, i.e., the lines overlap nearly before 200 km in Figure 1, Figure 2 and Figure 3. Also, we note that the SKR estimated with the method in this paper when without side channels is lower than that of [11,17,22] by comparing the dashed line with the solid line when θ=0 and ν=0 in Figure 3. Therefore, we could choose the original method [11,17,22] when without side channels and choose the method in this paper when with side channels.

## 6. Conclusions

In this paper, we take into account the imperfections at the light source and enhance the practical implementation of the SNS protocol. We analyze that the flaws of phase modulation can be overcome by regarding the deviation of the phase as phase noise and eliminating it with the method of the post-selection of phase. To overcome the side channels that may arise from THAs or device imperfections, we modify the GLT method [42] to the SNS protocol. Remarkably, we decompose the two-mode single-photon states into two special parts which are not the Pauli operators to reduce the number of parameters that need to be solved. To make it more practical, we analyze how to apply this method with the four-intensity decoy-state method. In this way, the practical implementations of the SNS protocol can be guaranteed without a detailed characterization of the side channels. Last, we note that the pulse correlations are not considered in this paper, which may be solved with the method in [28,33,34,36,50]. In conclusion, our results might constitute a crucial step towards guaranteeing the practical implementations of the SNS protocol with flawed and leaky sources.

## Figures and Tables

**Table 1:** List of experimental parameters about the active devices. Here, γ is the fiber loss coefficient (dB/km), $\eta_{d}$ is the detection efficiency of detectors, $e_{d}$ is the misalignment-error probability, $f_{\mathrm{EC}}$ is the error correction inefficiency, and $p_{d}$ is the dark count rate.

γ	$\eta_{d}$	$e_{d}$	$f_{\mathrm{EC}}$	$p_{d}$
0.2	56%	3%	1.1	$10^{-8}$

**Table 2:** List of experimental parameters about intensities and probabilities in different windows that Alice and Bob select. *M* is the number of phase slices [26].

$\mu_{a}$	$\mu_{b}$	$\mu_{z}$	$p_{z}$	$p_{a}$	$p_{b}$	$p_{z0}$	*M*
0.1	0.384	0.447	0.776	0.85	0.073	0.732	16

## Data Availability

Not applicable.

## Appendix A. Virtual Protocol

In this section, we introduce the virtual protocol based on [11]. Suppose Alice and Bob preshare the classical information for different windows and the specific intensities in *X* windows so there are no mismatching windows, e.g., the time window when one commits to the signal window but the other commits to the decoy window, or when they choose different intensities when both decide the decoy window. Equivalently, we could assume Alice and Bob are sitting in the same lab so they can agree on the windows and intensities. This restriction can be released since these windows are a subset of the actual protocol [11].

In *Z* windows, they prepare the following states

(A1) $$|\Psi_{z}^{q}\rangle_{CEC^{\prime }E^{\prime }AB}=\frac{1}{\sqrt{2}}{[|0\Phi_{z}\rangle }_{CEC^{\prime }E^{\prime }}⊗{|01\rangle }_{AB}+{\left(-1\right)}^{q}|\Phi_{z}{0\rangle }_{CEC^{\prime }E^{\prime }}⊗{|10\rangle }_{AB}],$$

where q∈{0,1}. Here, systems *A* and *B* are Alice and Bob’s ancillary system, respectively. Alice (Bob) can measure the ancillary state in the basis {|0〉,|1〉} and puts down bit 1 (0) and 0 (1) when obtaining state |1〉 and |0〉. In $X_{\alpha }$ (α∈{a,b}) windows, they prepare the state

(A2) $$|\Psi_{\alpha }^{\left(\theta_{A},\theta_{B}\right)}\rangle_{CEC^{\prime }E^{\prime }AB}=\frac{1}{\sqrt{2}}{[e^{i\theta_{B}}|0\Phi_{\alpha }\rangle }_{CEC^{\prime }E^{\prime }}⊗{|01\rangle }_{AB}+e^{i\theta_{A}}|\Phi_{\alpha }{0\rangle }_{CEC^{\prime }E^{\prime }}⊗{|10\rangle }_{AB}],$$

with phases $\theta_{A}$ and $\theta_{B}$ obeying unifrom distribution in [0,2π). The states sent in systems *C* and $C^{\prime }$ may be mixed due to the imperfections of their devices which could include a potential entanglement between their devices and Eve’s systems. Therefore, the mixed states in system *C* ($C^{\prime }$) in a single-mode qubit space can be purified by introducing Alice and Eve’ (Bob and Eve’) ancillary systems. Here, we assume that the mixed can be purified only by Eve’s ancillary systems *E* and $E^{\prime }$ for simplicity so that the formulas in Equations (A1) and (A2) could include mixed states in single-mode qubit space. Please note that this assumption is conservative since the purification is controlled by Eve completely. We note that it would not affect the security if we abandon this assumption.

Rewrite the state in Equation (A1) in the *X* basis of the systems *A* and *B* as

(A3) $$\begin{array}{cc} |\Psi_{z}^{q}\rangle_{CEC^{\prime }E^{\prime }AB}= & \frac{1}{\sqrt{2}}{|\varphi_{even}^{z,q,vir}\rangle }_{CEC^{\prime }E^{\prime }}\frac{|0_{x}0_{x}\rangle_{AB}-{|1_{x}1_{x}\rangle }_{AB}}{\sqrt{2}}\\ \; & +\frac{1}{\sqrt{2}}{|\varphi_{odd}^{z,q,vir}\rangle }_{CEC^{\prime }E^{\prime }}\frac{|1_{x}0_{x}\rangle_{AB}-{|0_{x}1_{x}\rangle }_{AB}}{\sqrt{2}}, \end{array}$$

where the normalized states

(A4) $$\begin{array}{c} |\varphi_{even}^{z,q,vir}\rangle_{CEC^{\prime }E^{\prime }}=\frac{1}{\sqrt{2}}{[|0\Phi_{\alpha }\rangle }_{CEC^{\prime }E^{\prime }}+{\left(-1\right)}^{q}|\Phi_{\alpha }0\rangle_{CEC^{\prime }E^{\prime }}],\\ |\varphi_{odd}^{z,q,vir}\rangle_{CEC^{\prime }E^{\prime }}=\frac{1}{\sqrt{2}}{[|0\Phi_{\alpha }\rangle }_{CEC^{\prime }E^{\prime }}-{\left(-1\right)}^{q}|\Phi_{\alpha }0\rangle_{CEC^{\prime }E^{\prime }}]. \end{array}$$

To obtain the phase error rate, Alice and Bob could measure the states in Equation (A2) of systems *A* and *B* in the *X* basis and then sift the results with phase satisfying Equations (1) and (2). Rewrite the state in Equation (A2) in the *X* basis of the systems *A* and *B* as

(A5) $$\begin{array}{cc} |\Psi_{\alpha }^{\left(\theta_{A},\theta_{B}\right)}\rangle_{CEC^{\prime }E^{\prime }AB}= & \frac{1}{\sqrt{2}}{|\varphi_{even}^{\alpha ,(\theta_{A},\theta_{B}),vir}\rangle }_{CEC^{\prime }E^{\prime }}\frac{|0_{x}0_{x}\rangle_{AB}-{|1_{x}1_{x}\rangle }_{AB}}{\sqrt{2}}\\ \; & +\frac{1}{\sqrt{2}}{|\varphi_{odd}^{\alpha ,(\theta_{A},\theta_{B}),vir}\rangle }_{CEC^{\prime }E^{\prime }}\frac{|1_{x}0_{x}\rangle_{AB}-{|0_{x}1_{x}\rangle }_{AB}}{\sqrt{2}}, \end{array}$$

where the normalized states

(A6) $$\begin{array}{c} |\varphi_{even}^{\alpha ,(\theta_{A},\theta_{B}),vir}\rangle_{CEC^{\prime }E^{\prime }}=\frac{1}{\sqrt{2}}{[e^{i\theta_{B}}|0\Phi_{\alpha }\rangle }_{CEC^{\prime }E^{\prime }}+e^{i\theta_{A}}|\Phi_{\alpha }0\rangle_{CEC^{\prime }E^{\prime }}],\\ |\varphi_{odd}^{\alpha ,(\theta_{A},\theta_{B}),vir}\rangle_{CEC^{\prime }E^{\prime }}=\frac{1}{\sqrt{2}}{[e^{i\theta_{B}}|0\Phi_{\alpha }\rangle }_{CEC^{\prime }E^{\prime }}-e^{i\theta_{A}}|\Phi_{\alpha }0\rangle_{CEC^{\prime }E^{\prime }}]. \end{array}$$

In the virtual protocol, Alice and Bob send the virtual states $|\varphi_{even}^{\alpha ,(\theta_{A},\theta_{B}),vir}\rangle_{CEC^{\prime }E^{\prime }}$, $|\varphi_{odd}^{\alpha ,(\theta_{A},\theta_{B}),vir}\rangle_{CEC^{\prime }E^{\prime }}$ (α∈{a,b}), $|\Psi_{z}^{q}\rangle_{CEC^{\prime }E^{\prime }AB}$, $|\varphi_{odd}^{z,q,vir}\rangle_{CEC^{\prime }E^{\prime }}$, and the actual state ${|00\rangle }_{CEC^{\prime }E^{\prime }}$, where ${|00\rangle }_{CEC^{\prime }E^{\prime }}$ is the states in *X* window which are used to estimate $Y_{0}$. Then by introducing the shield system $S_{A}$ and $S_{B}$, the selection of these actual and virtual states can be shown as

(A7) $${|\varphi \rangle }_{S_{A}S_{B}CEC^{\prime }E^{\prime }}=\sum_{l=1}^{13}\sqrt{P(l)}|l_{A}\rangle_{S_{A}}|l_{B}\rangle_{S_{B}}{|\vartheta_{l}\rangle }_{CEC^{\prime }E^{\prime }},$$

where

(A8) $$\begin{array}{cc} \; & |\vartheta_{1}\rangle_{CEC^{\prime }E^{\prime }}=|\vartheta_{2}\rangle_{CEC^{\prime }E^{\prime }}={|\varphi_{even}^{a,(\theta_{A},\theta_{B}),vir}\rangle }_{CEC^{\prime }E^{\prime }},\\ \; & |\vartheta_{3}\rangle_{CEC^{\prime }E^{\prime }}=|\vartheta_{4}\rangle_{CEC^{\prime }E^{\prime }}={|\varphi_{odd}^{a,(\theta_{A},\theta_{B}),vir}\rangle }_{CEC^{\prime }E^{\prime }},\\ \; & |\vartheta_{5}\rangle_{CEC^{\prime }E^{\prime }}=|\vartheta_{6}\rangle_{CEC^{\prime }E^{\prime }}={|\varphi_{even}^{b,(\theta_{A},\theta_{B}),vir}\rangle }_{CEC^{\prime }E^{\prime }},\\ \; & |\vartheta_{7}\rangle_{CEC^{\prime }E^{\prime }}=|\vartheta_{8}\rangle_{CEC^{\prime }E^{\prime }}={|\varphi_{odd}^{b,(\theta_{A},\theta_{B}),vir}\rangle }_{CEC^{\prime }E^{\prime }},\\ \; & |\vartheta_{9}\rangle_{CEC^{\prime }E^{\prime }}=|\vartheta_{9}\rangle_{CEC^{\prime }E^{\prime }}={|\varphi_{even}^{z,q,vir}\rangle }_{CEC^{\prime }E^{\prime }},\\ \; & |\vartheta_{11}\rangle_{CEC^{\prime }E^{\prime }}=|\vartheta_{12}\rangle_{CEC^{\prime }E^{\prime }}={|\varphi_{odd}^{z,q,vir}\rangle }_{CEC^{\prime }E^{\prime }},\\ \; & |\vartheta_{13}\rangle_{CEC^{\prime }E^{\prime }}={|00\rangle }_{CEC^{\prime }E^{\prime }}, \end{array}$$

with prababilities

(A9) $$\begin{array}{cc} \; & P\left(l\right)=\frac{1}{4}P_{x}P_{a},\;l=1,2,3,4,\\ \; & P\left(l\right)=\frac{1}{4}P_{x}P_{b},\;l=5,6,7,8,\\ \; & P\left(l\right)=\frac{P_{z}}{4},\;l=9,10,11,12,\\ \; & P\left(13\right)=P_{x}P_{v}. \end{array}$$

For l=1 to 12 we list $l_{A}l_{B}$ in order in the following, which corresponds to the measurement results they will obtain in the *X* basis

(A10) $$\{0_{x}0_{x},1_{x}1_{x},1_{x}0_{x},0_{x}1_{x},0_{x}0_{x},1_{x}1_{x},1_{x}0_{x},0_{x}1_{x},0_{x}0_{x},1_{x}1_{x},1_{x}0_{x},0_{x}1_{x}\}.$$

Denote the measurement result as vv for l=13 which corresponds to the events when both send |0〉 in decoy windows.

Here, as we do not consider the mismatching windows, we set

(A11) $$\begin{array}{cc} P_{x}= & \frac{p_{x}^{2}}{p_{x}^{2}+p_{z}^{2}},\\ P_{z}= & \frac{p_{z}^{2}}{p_{x}^{2}+p_{z}^{2}},\\ P_{a}= & \frac{p_{a}^{2}}{p_{a}^{2}+p_{b}^{2}+p_{v}^{2}},\\ P_{b}= & \frac{p_{b}^{2}}{p_{a}^{2}+p_{b}^{2}+p_{v}^{2}},\\ P_{v}= & \frac{p_{v}^{2}}{p_{a}^{2}+p_{b}^{2}+p_{v}^{2}}. \end{array}$$

In the following, we introduce the virtual protocol.

(1) State preparation. Alice and Bob prepare systems *S* (i.e., $S_{A}S_{B}$), CE and $C^{\prime }E^{\prime }$ in Equation (A7), and then send the systems CE and $C^{\prime }E^{\prime }$ to Charlie. They repeat this step *N* times.

(2) Interferometic measurement. Charlie does the same as Step 2 in the actual protocol in Section 2.

(3) Sifting and measurement. They announce their phases in $X_{\alpha }$ windows and sift the effective events as defined in the actual protocol but with two different phase slices, Δ denoted as $\Delta_{1}$ and $\Delta_{2}$. Then they measure their shield system $S_{A}$ and $S_{B}$ and obtain *l* for those effective events, and measure the systems *A* and *B* in *X* (*Z*) when l=1 to 12 (l=13).

(4) Announcement. They announce their measurement results when l=1 to 8 which are used to estimate the phase error rate with Charlie’s measurement results.

## Appendix B. Security Proof Against Coherent Attacks

In coherent attacks, Eve is allowed to interact with all states and then perform a joint measurement. For this, we could use Azuma’s inequality [42,51] to derive a relation between the expected and observed values which can include the dependencies.

Consider all the states Alice and Bob prepare in the whole process as

(A12) $${|\tau \rangle }_{SCEC^{\prime }E^{\prime }}=|\varphi_{k-1}\rangle_{SCEC^{\prime }E^{\prime }}|\varphi_{k}\rangle_{SCEC^{\prime }E^{\prime }}{|\varphi_{r}\rangle }_{SCEC^{\prime }E^{\prime }},$$

where $|\varphi_{k-1}\rangle_{SCEC^{\prime }E^{\prime }}$, $|\varphi_{k}\rangle_{SCEC^{\prime }E^{\prime }}$ and $|\varphi_{r}\rangle_{SCEC^{\prime }E^{\prime }}$ correspond to all the states before the *k*th runs, in the *k*the run and in the rest of the all runs, respectively. The system *S* represents the systems $S_{A}$ and $S_{B}$. Suppose Eve’s operation on all systems as

(A13) $${\hat{U}}_{CEC^{\prime }E^{\prime }E^{\prime \prime }}{|\tau \rangle }_{SCEC^{\prime }E^{\prime }}⊗{|0\rangle }_{E^{\prime \prime }}=\sum_{t}{\hat{B}}_{tCEC^{\prime }E^{\prime }}{|\tau \rangle }_{SCEC^{\prime }E^{\prime }}{|t\rangle }_{E^{\prime \prime }},$$

where ${\hat{U}}_{CEC^{\prime }E^{\prime }E^{\prime \prime }}$ is a unitary transformation on systems $CEC^{\prime }E^{\prime }E^{\prime \prime }$, and ${\hat{B}}_{tCEC^{\prime }E^{\prime }}$ is the Kraus operator acting on systems $CEC^{\prime }E^{\prime }$ depending on Eve’s measurement result *t*, and {|t〉} is an orthonormal basis. Here, system $E^{\prime \prime }$ is an ancillary system Eve prepares to operate and measure so as to obtain information. Let the joint operator

(A14) $${\hat{O}}_{k-1,SCEC^{\prime }E^{\prime }}=\overset{k-1}{\underset{v=1}{⨂}}{\hat{M}}_{S_{v}CEC^{\prime }E_{v}^{\prime }},$$

where ${\hat{M}}_{S_{v}CEC^{\prime }E_{v}^{\prime }}$ denotes the Kraus operator corresponds to the *v*th measurement results of the Alice, Bob and Charlie. Therefore, the normalized state of the *k*th systems $SCEC^{\prime }E^{\prime }$ conditioned on the measurement results ${\hat{O}}_{k-1}$ of the first k−1 systems can be shown as

(A15) $${\hat{\rho }}_{k|O_{k-1}^{SCEC^{\prime }E^{\prime }}}=\frac{{\hat{\sigma }}_{k|O_{k-1}}^{SCEC^{\prime }E^{\prime }}}{\mathrm{Tr}({\hat{\sigma }}_{k|O_{k-1}}^{SCEC^{\prime }E^{\prime }})},$$

where

(A16) $${\hat{\sigma }}_{k|O_{k-1}}^{SCEC^{\prime }E^{\prime }}=\sum_{t}\sum_{{\vec{x}}_{k-1},{\vec{x}}_{r}}A_{t,CEC^{\prime }E^{\prime }|k-1}^{\left({\vec{x}}_{k-1},{\vec{x}}_{r}\right)}|\varphi_{k}\rangle_{SCEC^{\prime }E^{\prime }}\langle \varphi_{k}|A_{t,CEC^{\prime }E^{\prime }|k-1}^{†\left({\vec{x}}_{k-1},{\vec{x}}_{r}\right)},$$

with

(A17) $$A_{t,CEC^{\prime }E^{\prime }|k-1}^{\left({\vec{x}}_{k-1},{\vec{x}}_{r}\right)}=\langle {\vec{x}}_{r}|\langle {\vec{x}}_{k-1}|{\hat{O}}_{k-1,SCEC^{\prime }E^{\prime }}{\hat{B}}_{tCEC^{\prime }E^{\prime }}{|\varphi_{k-1},\varphi_{r}\rangle }_{SCEC^{\prime }E^{\prime }},$$

and the basis $\{|{\vec{x}}_{k-1}\rangle ,|{\vec{x}}_{r}\rangle \}$, where $|{\vec{x}}_{k-1}\rangle$ corresponds to all the systems in the first k−1 runs and $|{\vec{x}}_{r}\rangle$ corresponds to the rest of the systems after *k*th run. Then by substituting Equation (A7) into Equation (A16), we obtain

(A18) $$\begin{array}{cc} {\hat{\sigma }}_{k|O_{k-1}}^{SCEC^{\prime }E^{\prime }}= & \sum_{l,l^{\prime }}\sqrt{P\left(l\right)P\left(l^{\prime }\right)}\\ \; & \times \sum_{t}\sum_{{\vec{x}}_{k-1},{\vec{x}}_{r}}A_{t,CEC^{\prime }E^{\prime }|k-1}^{\left({\vec{x}}_{k-1},{\vec{x}}_{r}\right)}|l_{A}l_{B}\rangle_{S}\langle l_{A}^{\prime }l_{B}^{\prime }\left|⊗\right|\vartheta_{l}\rangle_{CEC^{\prime }E^{\prime }}\langle \vartheta_{l^{\prime }}|A_{t,CEC^{\prime }E^{\prime }|k-1}^{†\left({\vec{x}}_{k-1},{\vec{x}}_{r}\right)}, \end{array}$$

Hence, the probability that Alice and Bob obtain $l_{A}l_{B}$ (l=1 to 8) in the *X* basis and only *d* detector clicks on Charlie’s side conditional on that all the previous measurement results and Alice and Bob’s phases satisfy Equation (1) or (2) (i.e., c=0 or 1) can be shown as

(A19) $$P_{l|O_{k-1}}^{(c,d),\Delta }=\frac{1}{\Delta }\int_{\pi \delta (c-1)-\Delta /2}^{\pi \delta (c-1)+\Delta /2}\frac{P(l)}{\mathrm{Tr}({\hat{\sigma }}_{k|O_{k-1}}^{SCEC^{\prime }E^{\prime }})}\mathrm{Tr}{({\hat{D}}_{d|O_{k-1}}|\vartheta_{l}\rangle }_{CEC^{\prime }E^{\prime }}\langle \vartheta_{l}|)d\left(\theta_{A}-\theta_{B}\right),$$

where

(A20) $${\hat{D}}_{d|O_{k-1}}=\sum_{t}\sum_{{\vec{x}}_{k-1},{\vec{x}}_{r}}A_{t,CEC^{\prime }E^{\prime }|k-1}^{†\left({\vec{x}}_{k-1},{\vec{x}}_{r}\right)}{\hat{M}}_{d}A_{t,CEC^{\prime }E^{\prime }|k-1}^{\left({\vec{x}}_{k-1},{\vec{x}}_{r}\right)},$$

represents Eve’s operation and Charlie’s measurement. The conditional probability $P_{l|O_{k-1}}^{(c,d),\Delta }/P\left(l\right)$ corresponds to the conditional probability in Equation (25) except that the conditions of the former probability include all previous measurement results. Please note that the parameters $k_{x}$, $m_{x}$ and α are indicated by the parameter *l*. Similarly, we can give the conditional probability for l=9 to 12 corresponds to Equation (13) as $P_{l|O_{k-1}}^{(q,d),\Delta }/P\left(l\right)$ where

(A21) $$P_{l|O_{k-1}}^{(q,d),\Delta }=\frac{P(l)}{\mathrm{Tr}({\hat{\sigma }}_{k|O_{k-1}}^{SCEC^{\prime }E^{\prime }})}\mathrm{Tr}{({\hat{D}}_{d|O_{k-1}}|\vartheta_{l}\rangle }_{CEC^{\prime }E^{\prime }}\langle \vartheta_{l}|),$$

In fact, we do not need the explicit form of ${\hat{D}}_{d|O_{k-1}}$ just as ${\hat{D}}_{d}$ in Section 3. With the actual yields corresponding to $Y_{k_{x},m_{x}}^{(c,d),\alpha ,\Delta }$ and the relationship between $Y_{k_{x},m_{x}}^{(q,d),vir}$ and $Y_{k_{x},m_{x}}^{(c,d),\alpha ,\Delta }$ which is similar to that in Section 3, we can obtain the bounds of the actual yields corresponding to $Y_{k_{x},m_{x}}^{(q,d),vir}$ by using Azuma’s inequality [42,51] twice. Therefore, the phase error rate can be obtained under coherent attacks.
